# Development of the Japanese version of the Preschool Confusion Assessment Method for the ICU

**DOI:** 10.1002/ams2.306

**Published:** 2017-09-15

**Authors:** Yujiro Matsuishi, Haruhiko Hoshino, Nobutake Shimojo, Yuki Enomoto, Takahiro Kido, Subrina Jesmin, Masahiko Sumitani, Yoshiaki Inoue

**Affiliations:** ^1^ Department of Emergency and Critical Care Medicine Faculty of Medicine University of Tsukuba Tsukuba Ibaraki Japan; ^2^ Pediatric Intensive Care Unit University of Tsukuba Hospital Tsukuba Ibaraki Japan; ^3^ Department of Pediatrics University of Tsukuba Hospital Tsukuba Ibaraki Japan; ^4^ Faculty of Health and Sports Science University of Tsukuba Tsukuba Ibaraki Japan; ^5^ Department of Anesthesiology and Pain Relief Center University of Tokyo Hospital Tokyo Japan

**Keywords:** back translation, delirium, Japanese version, pediatric delirium, psCAM‐ICU

## Abstract

**Aim:**

Delirium is associated with various negative clinical outcomes, such as decline in cognitive ability, increased length of hospital stay, and higher mortality. For these reasons, early diagnosis of delirium is critical. Unfortunately, there are no reliable diagnostic criteria or tool of delirium for infants and preschool‐aged children in Japan.

The aim of the present study was to translate a new delirium assessment tool, the Preschool Confusion Assessment Method for the Intensive Care Unit (psCAM‐ICU), for accurately diagnosing clinically ill infants and preschool‐aged children, from English to Japanese.

**Methods:**

The translation was undertaken with the internationally established back‐translation method. The translation was repeated blindly and independently by eight medical researchers and clinicians from multiple disciplines. Any discrepancy evident from the translated works was discussed and resolved.

**Results:**

We report the successful development of the Japanese version of psCAM‐ICU. However, before its full application, this diagnostic tool requires further testing and study, most notably for its validation and reliability.

**Conclusion:**

A Japanese version of the psCAM‐ICU was developed.

## Introduction

Delirium is a common neuropsychological complication observed in adult patients in the intensive care unit (ICU) and is associated with negative clinical outcomes, such as higher mortality,^1^ a decline in cognitive abilities,^2^ and increased length of hospital stay, as well as re‐admission.^2^ For these reasons, the early diagnosis and effective monitoring of delirium is very critical. Currently, the most reliable assessment tool for diagnosing delirium is called the Confusion Assessment Method for the ICU.^3^ It is used to diagnose delirium in adults in ICUs, and has already been translated into Japanese. However, no reliable assessment tool for diagnosing pediatric delirium, such as the Preschool Confusion Assessment Method for the ICU (psCAM‐ICU),^4^ has been translated into Japanese. The psCAM‐ICU is in agreement with the recent standard delirium diagnosis, Diagnostic and Statistical Manual of Mental Disorders, 5th edition, and reportedly has high reliability and validity for ill infant and preschool‐aged children.^4^ Thus, the aim of the present study was to translate the psCAM‐ICU using the back‐translation method.

## Method

**Figure 1:**
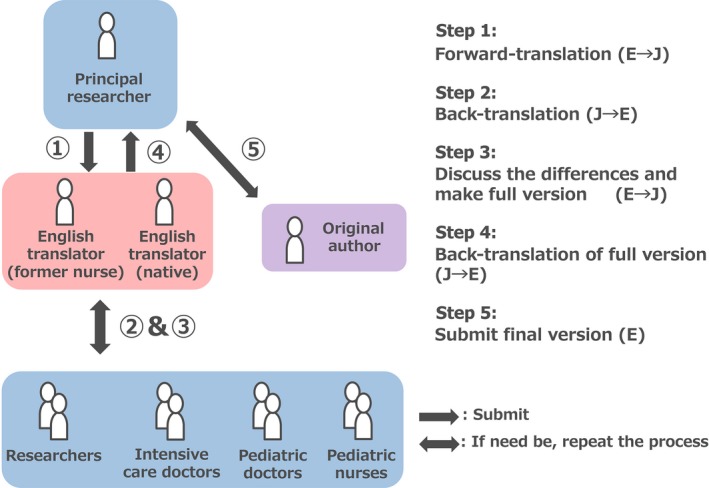
Flow of the back translation method used to translate the Preschool Confusion Assessment Method for the Intensive Care Unit from English (E) to Japanese (J). Steps 3 and 4 were repeated once in this study.

Prior to the commencement of the project, permission to translate the psCAM‐ICU was obtained from the developer, Dr. Heidi A.B. Smith. Translation was carried out using the back‐translation method,^5^ a well‐known method that ensures preservation of the overall content and meaning between the original and translated versions. In this translation process (Fig. 1), the principal researcher first translated the assessment tool from English to Japanese, generating a provisional Japanese version. In the second step, we submitted the manuscripts to a second set of translators, including a Japanese nurse who had worked in the USA and a native speaker of English (American). The translators back‐translated the manuscript. In the third step, eight medical professionals (including two clinical researchers, two intensive care doctors, two pediatric doctors, and two pediatric intensive care nurses) discussed the differences between the provisional Japanese version and the back‐translated version. These medical professionals then generated a revised provisional Japanese version. In the fourth step, the translators back‐translated the revised provisional Japanese version from Japanese to English. For consistency in translation as well as reduction in variability between multidisciplinary medical staff, the local medical professionals carefully checked any possible differences between the original and back‐translated versions. Every effort was made to carefully execute all the steps in order to avoid the loss of the original content due to cultural differences. After all of these steps, the final document was then checked by the original author, and the first author visited the original author's hospital (The Monroe Carell Jr. Children's Hospital, Vanderbilt, Nashville, TN, USA) to discuss the procedure of psCAM‐ICU.

## Results

**Figure 2:**
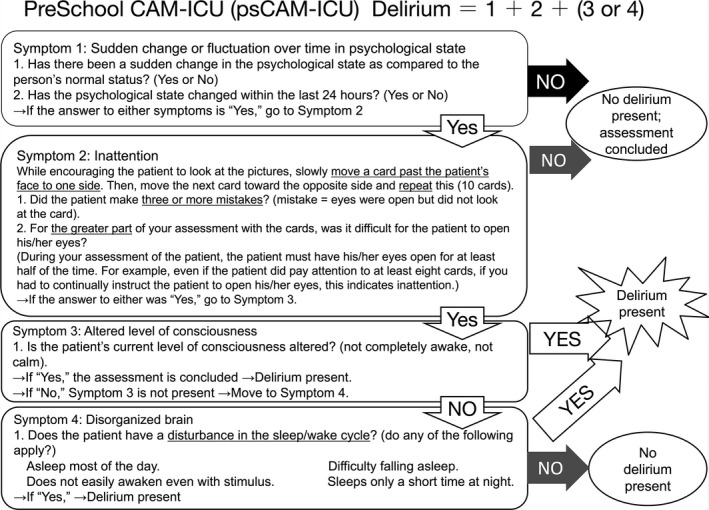
Final back‐translated English version of the Preschool Confusion Assessment Method for the Intensive Care Unit (psCAM‐ICU). This document was checked by the original author (Dr Heidi Smith).

The third and fourth steps described above were repeated once and then checked by the original author. Here, we show the completed versions of both back‐translated documents, that is, the English psCAM‐ICU (Fig. 2) and Japanese psCAM‐ICU manuscripts (Fig. S1). Some of the major changes made between the first and second translations are words such as those associated with “disorganized brain”. Our initial impression was that the translation or meaning of the word “brain” had to do with “thinking”. However, after a careful examination of this assessment point (Fig. 2, Symptom 4: Disorganized brain), the phrase had to do with the sleep–wake cycle and not “thinking”, which is not normally considered in assessing a 6‐month‐old child. Therefore, we replaced the initial word with a term that focused on “function”. After consulting the author, the author advised that we add the word “AND” between partial wakefulness or alertness and lack of calmness. The rational for this adjustment is that some clinicians describe patients with decreased levels of consciousness as calm. The ideal for a clinician requiring a response from a patient is a condition where the patient is in a state of alertness as well as calmness. Therefore, we added “AND” between “not completely awake” and “not calm”, per author's advice.

## Discussion

In the present study, The Japanese version of psCAM‐ICU was developed by using the back‐translation method. Generally speaking, a direct translation of a given assessment or diagnostic tool does not necessarily guarantee equivalence.^6^ The back‐translation method is a more valid method as it maintains the linguistic equivalency, and for this reason Brislin's classic back translation has often been used. Nonetheless, back translation on its own without considering cultural differences has its limitations in translation.^7^ Therefore, we chose to reference Jones’ back‐translation method,^5^ which involves multiple people in the process of translation. In addition, in the present study, we used participants from multiple medical disciplines and cultures in order to reduce the cultural differences. Further studies are needed in future to test the validation and reliability of the newly translated Japanese version of psCAM‐ICU.

## Conclusion

We developed the Japanese version of the psCAM‐ICU using a standardized procedure.

## Disclosure

Tsukuba University Hospital's institutional review board approved this study (approval no. H28‐085).

Conflict of Interest: Authors declare no Conflict of Interests for this article.

## Supporting information


**Fig. S1** Japanese version of the Preschool Confusion Assessment Method for the Intensive Care Unit (psCAM‐ICU), developed using the back‐translation method. To reduce variability between multidisciplinary medical staff, eight local medical workers carefully checked any possible differences between the original and back‐translated versions.
